# Dapagliflozin alleviates renal fibrosis in a mouse model of adenine-induced renal injury by inhibiting TGF-β1/MAPK mediated mitochondrial damage

**DOI:** 10.3389/fphar.2023.1095487

**Published:** 2023-03-07

**Authors:** Jianhua Zeng, Hao Huang, Yan Zhang, Xin Lv, Jiawei Cheng, Si Jue Zou, Yuanyuan Han, Songkai Wang, Li Gong, Zhangzhe Peng

**Affiliations:** ^1^ Department of Nephrology, Xiangya Hospital, Central South University, Changsha, China; ^2^ Hunan Key Laboratory of Organ Fibrosis, Central South University, Changsha, China; ^3^ National Clinical Research Center for Geriatric Disorders, Xiangya Hospital, Central South University, Changsha, China; ^4^ Department of Cell Biology, School of Life Sciences, Central South University, Changsha, China

**Keywords:** renal fibrosis, dapagliflozin, mitochondrial damage, TGF-β1/MAPK pathway, oxidative stress

## Abstract

Renal fibrosis is a common pathological outcome of various chronic kidney diseases, and as yet, there is no specific treatment. Dapagliflozin has shown renal protection in some clinical trials as a glucose-lowering drug, but its role and mechanism on renal fibrosis remain unclear. In this study, we used a 0.2% adenine diet-induced renal fibrosis mouse model to investigate whether dapagliflozin could protect renal function and alleviate renal fibrosis in this animal model. *In vivo*, we found that dapagliflozin’s protective effect on renal fibrosis was associated with 1) sustaining mitochondrial integrity and respiratory chain complex expression, maintained the amount of mitochondria; 2) improving fatty acid oxidation level with increased expression of CPT1-α, PPAR-α, ACOX1, and ACOX2; 3) reducing inflammation and oxidative stress, likely *via* regulation of IL-1β, IL-6, TNF-α, MCP-1, cxcl-1 expression, and glutathione (GSH) activity, superoxide dismutase (SOD) and malondialdehyde (MDA) levels; and 4) inhibiting the activation of the TGF-β1/MAPK pathway. In HK2 cells treated with TGF-β1, dapagliflozin reduced the expression of FN and α-SMA, improved mitochondrial respiratory chain complex expression, and inhibited activation of the TGF-β1/MAPK pathway.

## 1 Introduction

Renal fibrosis is a common pathological outcome of various chronic kidney diseases (CKD), which affects about 13% of the world’s human population (Zhu et al., 2021; Evans et al., 2022). Except for the management of existing comorbidities, such as diabetes, hypertension and obesity, there remains no effective therapy for CKD and renal fibrosis due to its complicated and ambiguous pathogenesis (Liu and Zhuang, 2019; Akinnibosun et al., 2022; Cao et al., 2022).

Diabetic nephropathy (DN) is a major cause of CKD that accounts for 30%–47% cases, some new drugs such as DPP-4 Inhibitors (sitagliptin, vildagliptin, saxagliptin, alogliptin and linagliptin, and preclinical), GLP-1 analogues (exenatide, liraglutide), sodium glucose cotransporter-2 (SGLT2) inhibitor (dapagliflozin, canagliflozin and empagliflozin) and so on show renal protection in DN (Schernthaner et al., 2014; Sharma et al., 2017; Sharma et al., 2018a; Kelly et al., 2019). Dapagliflozin is a potent, reversible, and selective inhibitor of (SGLT2), which is currently approved for type 2 diabetes treatment by the European Union; it is also the first SGLT2 inhibitor approved for using in type 1 diabetes (Dhillon, 2019; Paik and Blair, 2019). Several well-designed clinical studies have shown that dapagliflozin has a significant protective effect on the renal and cardiovascular systems, in addition to robust control of blood sugar levels (Wiviott et al., 2019; Kurata and Nangaku, 2022). In a dapagliflozin and CKD outcome prevention (DAPA-CKD) trial, dapagliflozin also reduced the risk of progressive kidney disease in non-diabetic chronic kidney disease (Persson et al., 2021). In basic diabetes-related research, dapagliflozin was found to reduce renal fibrosis in a type I and type II diabetic nephropathy mouse model (Terami et al., 2014; Tang et al., 2017; Huang et al., 2019). Due to its protective effect in the kidneys of patients with non-diabetic chronic kidney disease, research into the use of dapagliflozin to treat renal fibrosis is underway. Studies have shown that dapagliflozin can reduce renal fibrosis in alcohol-induced kidney injury and unilateral ureteral obstruction (UUO) animal models (Wu et al., 2021; Xuan et al., 2021; Liu et al., 2022).

Adenine, the basic material for DNA and RNA synthesis in the body, is widely used to treat leukopenia (Tomita et al., 2016). However, excessive intake of adenine can lead to high levels of accumulation in the kidneys, which can lead to severe nephrotoxicity, destruction of the renal structure, and ultimately renal fibrosis (Tamura et al., 2009; Boon et al., 2015). The present model simulates a genetic deficiency of human adenine phosphate glycosyltransferase (APRT), which causes 2,8-dihydroxy adenine (2,8-DHA) nephropathy, a rare disease characterized by formation of 2,8-dihydroxy adenine (2,8-DHA) in the renal tubules. The resulting crystals often damage the renal structure and impair renal function, leading to kidney failure (Runolfsdottir et al., 2016; Klinkhammer et al., 2020). This model has also been widely used to study renal fibrosis (Vázquez-Méndez et al., 2020; Yi et al., 2021; Ito et al., 2022), however, the effect of dapagliflozin on renal fibrosis induced by a high-adenine diet remains unclear.

In this study, we aimed to investigate the protective effect of dapagliflozin in a renal fibrosis mouse model induced by a 0.2% adenine diet and to explore the underlying pharmacological mechanisms, then provide a more adequate theoretical basis for dagliflozin in the treatment of renal fibrosis.

## 2 Methods

### 2.1 Reagents and materials

Dapagliflozin was obtained from Apexbio (United States, ^#^A5854), with a purity of ≥99%; TGF-β1was obtained from PeproTech (United States). The western blot antibodies used were Collagen 1 (Abcam, United States, ^#^ab270993), α-SMA (Sigma, United States, ^#^A5691), vimentin (Proteintech, United States, ^#^10366-1-AP), VDAC1(Proteintech, United States, ^#^55259-1-AP), FN (Proteintech, United States, ^#^15613-1-AP), p-JNK (CST, United States, ^#^4668), JNK (CST, United States, ^#^9252), p-ERK (CST, United States, ^#^4370), ERK (CST, United States, ^#^4695), P-P38 (CST, United States, ^#^4631), p38 (CST, United States, ^#^8690), total oxphos complexes (Thermo, United States, AB-2533835), CPT1-α (Proteintech, United States, ^#^15184-1-AP), 0.2% adenine diet (TP 1S002), and control diet (LAD 3001) obtained from Trophic Animal Feed High-tech Co., Ltd., China. The SOD activity kit (^#^A001-3), GSH content assay kit (^#^A006-2-1) and MDA content assay kit (^#^A003-1) were purchased from the Nanjing Jiancheng Institute of Bioengineering.

### 2.2 Animal model and treatment

Eight-week-old male C57BL/6J mice were obtained from Silaike Laboratory (Hunan, China) and fed in the animal experiment laboratory of Central South University with approval from the Animal Care and Use Committee of Central South University. After strict adaptive feeding for 1 week, mice were divided into four groups: normal diet with vehicle control (oral gavage, 0.1 mL/10 g, *n* = 6), 0.2% adenine diet with vehicle control (oral gavage, 0.1 mL/10 g, *n* = 8), 0.2% adenine diet with dapagliflozin therapy at a dose of 1 mg/kg/day (oral gavage, 0.1 mL/10 g, *n* = 8) (Wang et al., 2021), and 0.2% adenine diet with dapagliflozin therapy at a dose of 10 mg/kg/day (oral gavage, 0.1 mL/10 g, *n* = 8) (Huang et al., 2019). During this study, body weight and food consumption of the four groups were recorded. On day 21 of the experiment, the mice were anesthetized *via* intraperitonaeal injection of pentobarbital (1%, 50 mg/kg) to harvest the kidneys and blood for further study.

### 2.3 Preparation of dapagliflozin

Dapagliflozin was fully dissolved in DMSO (Sigma Aldrich, United States, ^#^D2650), according to the manufacturer’s instructions, and stored at −20°C. When needed, it was diluted in normal saline to a final concentration of DMSO of 1%. Dapagliflozin was administered when mice were fed a 0.2% adenine diet.

### 2.4 Serum creatinine, urea nitrogen, and uric acid

Blood samples obtained from the previous animal experiments were placed in an incubator at 37°C for 1 h. After centrifugation at 4°C and 3,000 rpm for 10 min, the serum was aspirated and sent to the laboratory of Xiangya Hospital of Central South University for detection of serum creatinine and urea nitrogen.

### 2.5 Histological examination

Tissue fixative was used to fix the isolated kidney, dehydrated, and embedded in paraffin. The paraffin sections were stained with hematoxylin-eosin to enable visualization of the kidneys pathological morphology, and the sections were stained with Masson’s trichrome to facilitate observation of the degree of renal fibrosis. Each kidney sample was randomly selected from 10 regions (200×) under a light microscope (Nikon, Ni Ci E100 E200, Japan) for semi quantitative analysis, and the grading was 0–4 (0: 0%; 1: <25%; 2: 26%–50%; 3: 51%–75%; 4: ≥76% of the damaged renal tubules), and ImageJ software (Image-Pro Plus 6.0 software, Media Cybernetics, Bethesda, MD, United States) was used for measurement and calculation (Ren et al., 2021). Frozen sections were prepared, and the kidney tissues were stained with Oil Red O dye for the observation of lipid deposition in the kidney tissues, the samples were examined using microscope (Nikon, Ni Ci E100 E200, Japan), and the images were analyzed using ImageJ software (Image-Pro Plus 6.0 software, Media Cybernetics, Bethesda, MD, United States) (Chen et al., 2021).

### 2.6 RNA-Seq Transcriptomic assay

Total RNA in kidney tissue was extracted using TRIzol (Invitrogen, United States, ^#^15596026), and the quality of total RNA was evaluated with an RNA6000 Nano LabChip kit (Agilent, California, United States) using the Agilent biological analyzer 2100 system. RNA sequencing was conducted by Beijing Berry Hekang Biotechnology Co., Ltd. EdgeR was used to analyze the significance of the differential expression. The actual analysis parameters used in this analysis were log_2_ | FoldChange |>2.0 and a q value of <0.05. Gene Ontology (GO) software was used for GO enrichment analysis of differentially expressed genes, and the KOBAS (v3.0) software was used for Kyoto Encyclopedia of Genes and Genomes (KEGG) enrichment analysis.

### 2.7 Transmission electron microscopy

The kidney tissue was first fixed with electron microscope fixative (Pinofet, S191102), followed by 1% osmium tetroxide, and was then dehydrated in graded alcohol and acetone and prepared into ultrathin sections (60–80 nm) in pure 812 embedding agent (SPI, 90529-77-4). The sections were stained with uranyl acetate and lead citrate, dried. Using transmission electron microscope (Hitachi; HT7800/HT7700), the number and size of mitochondria were measured in 20 random unoverlapped proximal tubular cells of each sample (Xuan et al., 2021).

### 2.8 Cell culture and intervention

Human proximal renal tubular epithelial cells (HK2) were purchased from Zhongqiao Xinzhou (China, ZQ0313). DMEM/F12 (Gbico, United States, ^#^11330032) containing 10% fetal bovine serum (FBS) (Gbico, United States, ^#^10099-141) and 1% penicillin-streptomycin was used to culture the cells in a cell culture box containing 5% carbon dioxide at 37°C. When the cell density was above 90%, 0.05% tryptase was used to digest the cells. The cell suspension obtained was terminated with DMEM/F12 containing FBS. Firstly, the cells from the 3×10^4^/well were inoculated into a 96 well plate. When the cells adhered to the wall and grew to 70% confluence, different concentrations of dapagliflozin were added. After 24 h of action, the CCK8 kit (Apexbio, United States, ^#^K1018) instructions were strictly followed to explore the effects of the drugs on normal cells. Cells were selected in good growth conditions and inoculated 5×10^5^ cells per well into a 12 well plate, then divided into a normal group, TGF-β1 (10 ng/mL) stimulation group, and dapagliflozin treatment group. TGF-β1 was given to both the stimulation group and the treatment group when the cell growth was 70%, and dapagliflozin was added to the treatment group. The principle behind selecting the treatment concentration was to select 1 or 2 concentrations that had no effect on normal cells. Cell proteins were obtained for subsequent analysis after 24 h of stimulation.

### 2.9 Western blotting

Sodium dodecyl sulfate (SDS) contained 1% protease inhibitor and 1% phosphatase inhibitor to lyse an appropriate amount of each kidney sample and cell sample. Cells were centrifuged at 13000 rpm at 4°C for 10 min. The supernatant was collected after the completion of lysis, and bicinchoninic acid (BCA) protein detection kit was used to quantify the obtained protein samples (Sharma et al., 2020). According to the indicators to be analyzed, 8%–12% polyacrylamide gel to electrophoresis was added to the obtained protein samples, which were subsequently transferred to a polyvinylidene fluoride (PVDF) membrane under a condition of 260 mA current for 90 min. The rapid sealing solution was applied for 15 min and the corresponding primary antibody was incubated at 4°C overnight. The next day, after incubation with the corresponding secondary antibody for 1 h, the membrane was imaged with a super-sensitivity developer or an ordinary sensitivity developer. ImageJ software was used to analyze the strip produced by the development.

### 2.10 RNA extraction and real-time quantitative PCR

Trizol was used to extract total RNA from kidney tissues. A Revert Aid First Strand cDNA Synthesis Kit (Thermo Scientific, MA, ^#^K1622) was used to reverse-transcribe RNA into DNA. Real-time PCR was performed using the CFX96 Quantitative PCR Detection System (Bio-rad, United States, ^#^1855200). The primer sequences used in this study are shown in Supplementary Table S1.

### 2.11 SOD, GSH, and MDA assays

To detect SOD, GSH and MDA content in the kidney tissues of mice in each group, kidney tissues stored in liquid nitrogen with appropriate weight were weighed and ground into 10% tissue homogenate with normal saline. We strictly followed the instructions to detect the activity of SOD the content of GSH and MDA in the kidney tissue (Sharma et al., 2018b; Fernandes et al., 2018; Sharma et al., 2019).

### 2.12 Intracellular ROS assay

Intracellular ROS was determined by 2′,7′-dichlorofluorescein diacetate (DCFH-DA) (Solarbio, ^#^4091-99-0) according to the manufacturer’s instructions. Briefly, HK-2 cells were seeded in 24-well plates. After 24 h incubation, cells were pretreated with or without various concentrations of dapaliglozin (20 and 40 μM) and coincubated with or without H_2_O_2_ (300 μmol/L) for 24 h (Zhang et al., 2019). Then HK-2 cells were loaded with 5 μmol/L DCFH-DA for 30 min at 37°C in the dark and washed with PBS three times. The cells were digested for analysis using a microplate reader at 485/528 nm (Bio-tek Synergy HT, United States).

### 2.13 Statistical analysis

All data are expressed as mean ± standard deviation. Comparisons between groups were analyzed using one-way ANOVA, and comparisons between two groups were analyzed using the least significant difference test. *p* < 0.05 was considered statistically significant.

## 3 Results

### 3.1 Dapagliflozin protected renal function and alleviated renal fibrosis in mice fed a 0.2% adenine diet

**FIGURE 1:**
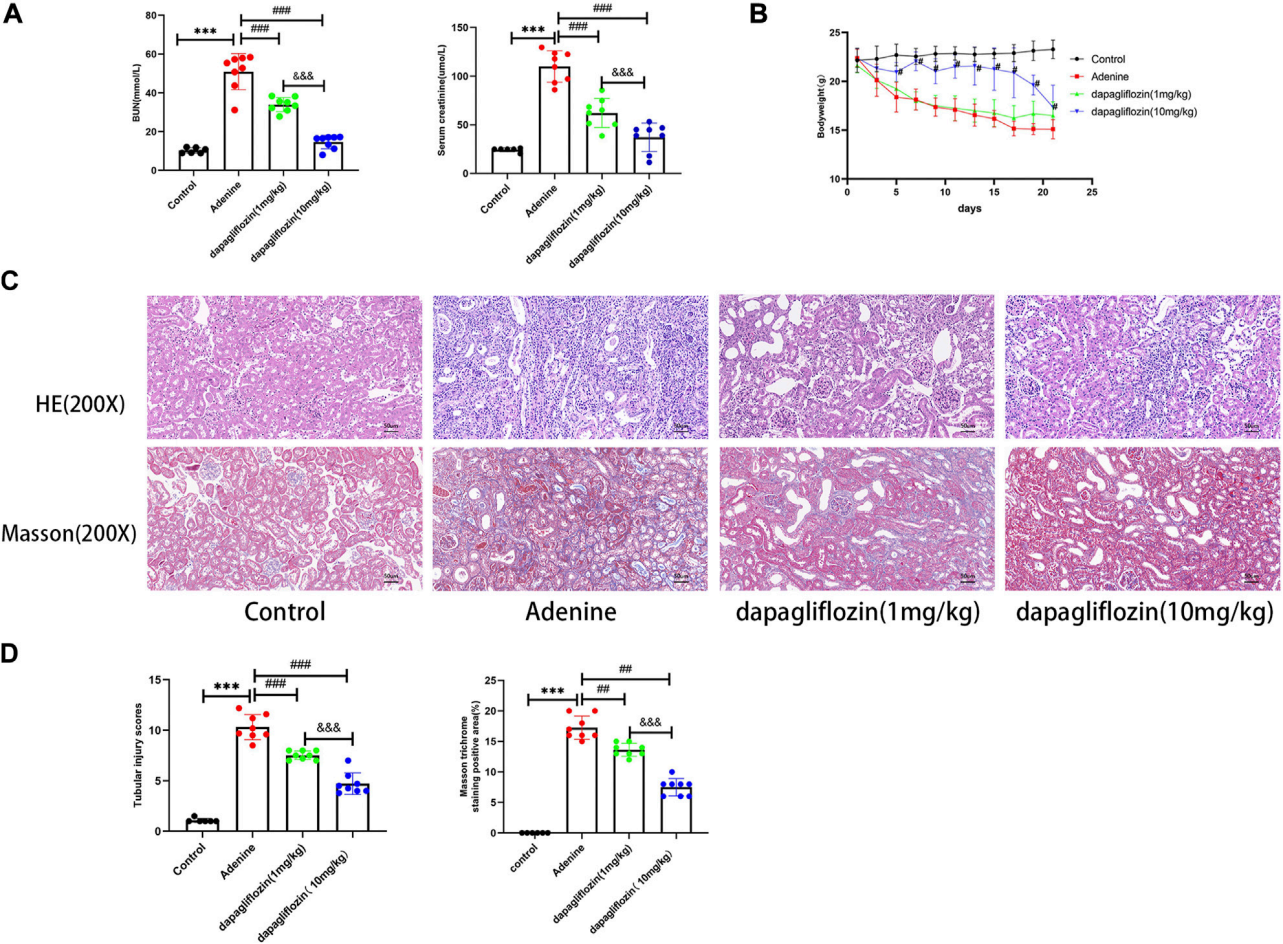
Dapagliflozin reduced renal fibrosis in 0.2% adenine diet-fed mice. **(A)** Serum urea nitrogen (BUN) and creatinine levels in each group. **(B)** Body weight variation of mice in each grough. **(C)** HE and Masson staining of renal tissues from each group. **(D)** Renal HE and Masson scores of each group. Scale bars, 50 μm for all groups. Values are means ± SD. ***p < 0.001 adenine diet group compared with control group; ^##^ *p* < 0.01 dapagliflozin treatment group compared with adenine diet group; ^###^ *p* < 0.001 dapagliflozin treatment group compared with adenine diet group; *p* < 0.001 1 mg/kg dapagliflozin treatment group compared with 10 mg/kg dapagliflozin treatment group.

Serum levels of urea nitrogen (BUN) and creatinine in 0.2% adenine diet-fed mice were significantly higher than those in control mice, the serum levels of urea nitrogen and creatinine were significantly decreased, and dapagliflozin at a dosage of 10 mg/kg was better than the dosage of 1 mg/kg (Figure 1A). During the period of the experiment, we found that the mice treated with 0.2% adenine had a significantly reduced body weight, dapagliflozin treatment was effective in reducing their weight loss, and the dosage of 10 mg/kg was better than the dosage of 1 mg/kg (Figure 1B). Hematoxylin and eosin (HE) and Masson staining were used to evaluate tubulointerstitial injuries and interstitial extracellular matrix (ECM) deposition, respectively. HE and Masson staining showed that the tubules in the kidneys of mice fed with a 0.2% adenine diet were markedly dilated and atrophied, and ECM deposition was observed in the renal interstitium, whereas mice treated with dapagliflozin showed a partial but significant decrease in both renal tubular injury and interstitial ECM deposition (Figure 1C). The HE and Masson pathological scores were higher in the 0.2% adenine diet mice than control group. After dapagliflozin treatment, the pathological scores in dapagliflozin treatment group were lower than the 0.2% adenine diet mice, indicating that dapagliflozin alleviated tubulointerstitial injuries and interstitial ECM deposition, and the higher dosage (10 mg/kg) was better than the lower dosage (1 mg/kg) (Figure 1D).

### 3.2 Dapagliflozin reduces the expression of fibrotic molecules

**FIGURE 2:**
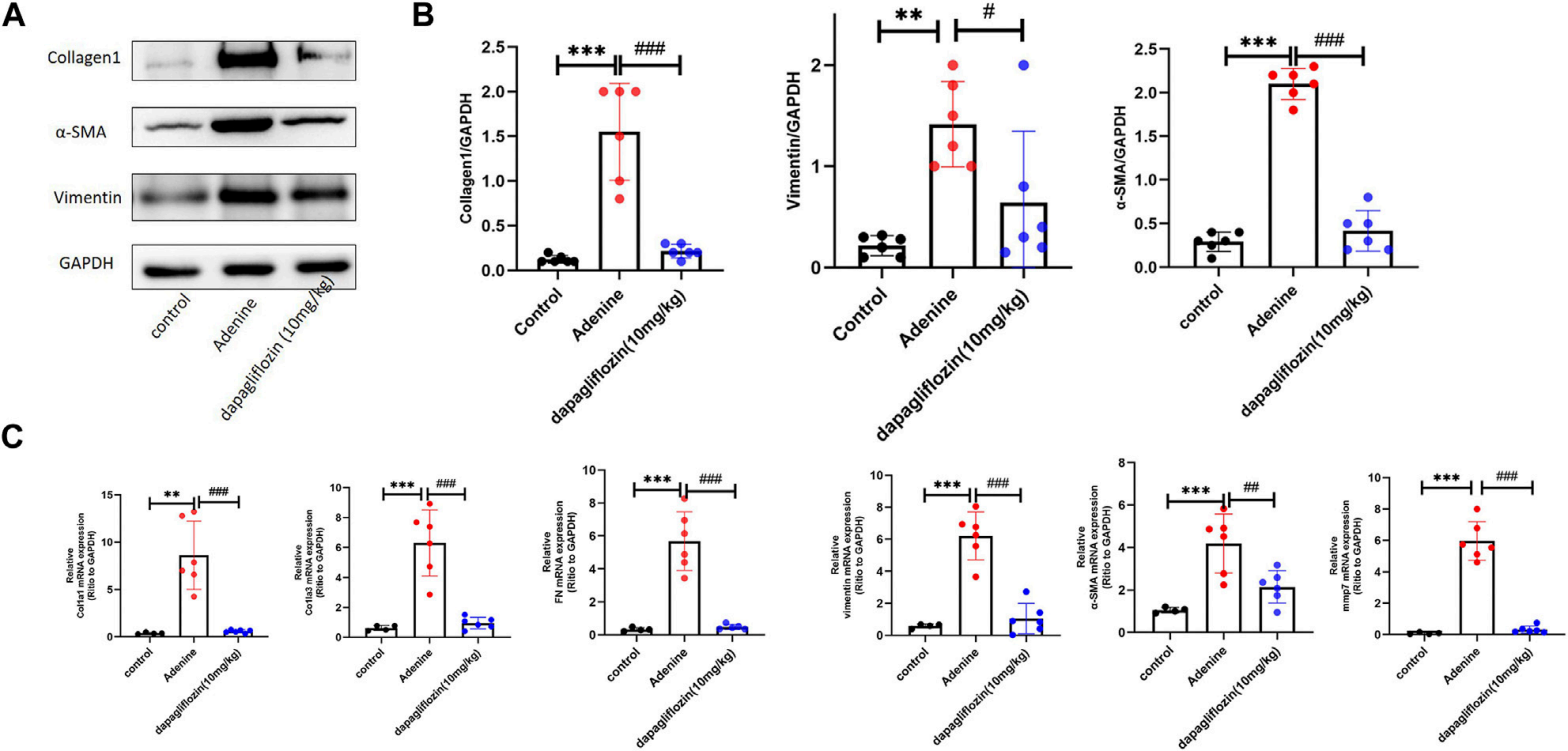
Dapagliflozin reduced the expression of renal fibrosis-related markers in 0.2% adenine diet-fed mice. **(A, B)** Western blot showing the expression of Collagen 1, α-SMA, and vimentin in the renal tissue of each group. **(C)** Real time PCR showing the expression of col1a1, col3a1, FN, Vimentin, α-SMA, and MMP7 in the renal tissue of each group. Values are means ± SD. **p < 0.01 adenine diet group compared with the control group; ***p < 0.001 adenine diet group compared with the control group; ^#^ *p* < 0.01 dapagliflozin treatment group compared with the adenine diet group; ^##^ *p* < 0.01 dapagliflozin treatment group compared with the adenine diet group; ^###^ *p* < 0.001 dapagliflozin treatment group compared with the adenine diet group.

As the effect of dapagliflozin at a dosage of 10 mg/kg was better than the dosage of 1 mg/kg, we used this dosage in further studies. Western blotting showed that proteins such as α-SMA, Vimentin and Collagen 1 were significantly upregulated in the kidneys of mice fed a 0.2% adenine diet, and significantly downregulated by 10 mg/kg dapagliflozin treatment (Figures 2A, B). Simultaneously, real-time Quantitative Polymerase Chain Reaction (PCR) confirmed that the expression of fibrotic genes, such as col1a1, col1a3, FN, α-SMA, and vimentin, was significantly upregulated in the kidneys of mice fed 0.2% adenine, and their expression was significantly downregulated by dapagliflozin treatment (Figure 2C).

### 3.3 Analysis of renal transcriptome in 0.2% adenine diet-induced renal fibrosis mice

**FIGURE 3:**
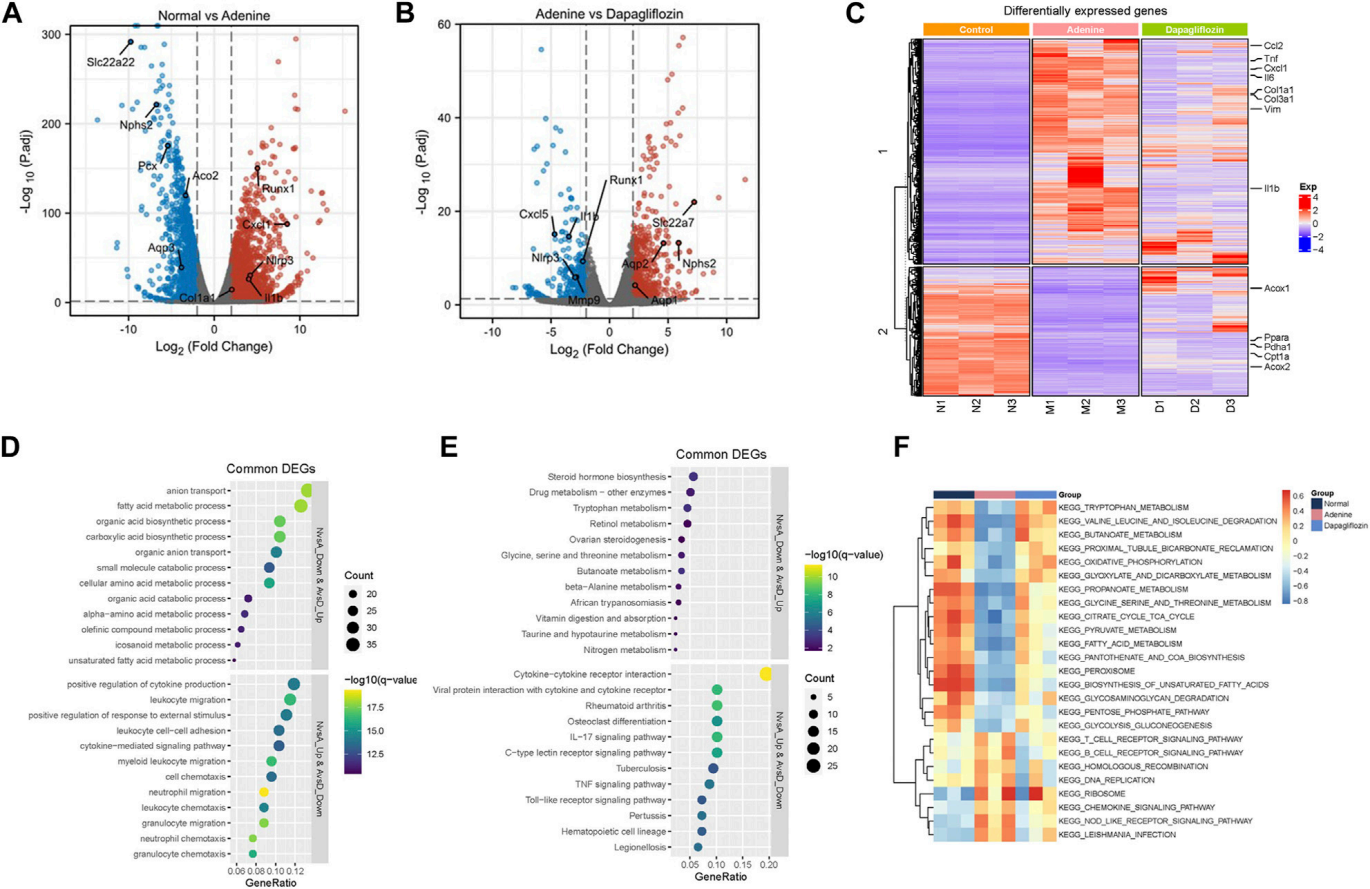
Analysis of renal transcriptome in 0.2% adenine diet-induced renal fibrosis mice. **(A)** Volcano plot of gene expression difference between the control and 0.2% adenine diet groups. **(B)** Volcano plot of gene expression difference between the 0.2% adenine diet and 10 mg/kg dapagliflozin treatment groups. **(C)** Heatmap of gene expression difference among the control, 0.2% adenine diet, and dapagliflozin 10 mg/kg groups. **(D)** GO analysis of differentially expressed genes. **(E, F)** KEGG analysis of differentially expressed genes. N: control diet M: 0.2% adenine diet D 0.2% adenine diet + dapagliflozin (10 mg/kg).

RNA-seq analysis was performed to understand how dapagliflozin alleviates renal fibrosis. The volcano plot shows significantly different gene expression profiles among normal diet mice, 0.2% adenine mice, and dapagliflozin (10 mg/kg)-treated mice. Among these differentially expressed genes, 2459 genes were upregulated, and 1357 genes were downregulated in the kidneys of 0.2% adenine diet-fed mice compared with control mice (*p* < 0.05) (Figure 3A). However, dapagliflozin (10 mg/kg) reversed the changes in 494 downregulated and 323 upregulated genes (*p* < 0.05) (Figure 3B). Significant dapagliflozin-modulated genes are illustrated by a heatmap (Figure 3C). Genes related to fibrosis (col1a1, col1a3) and inflammation (TNF-α, IL-6, IL-1β) were upregulated in 0.2% adenine diet mice, and were downregulated by dapagliflozin (10 mg/kg) (Figure 3C). The heatmap also showed that mitochondrial metabolism and fatty acid oxidation (PDH, Acox1, and Acox2)-related genes were downregulated in 0.2% adenine diet-fed mice and upregulated by dapagliflozin (Figure 3C). Furthermore, GO (Figure 3D) and KEGG (Figures 3E, F) enrichment analyses suggested that these differentially expressed genes were involved in mitochondrial and lipid metabolism and inflammatory responses.

### 3.4 Dapagliflozin increased mitochondrial metabolism and fatty acid oxidation in the kidneys of mice fed 0.2% adenine

**FIGURE 4:**
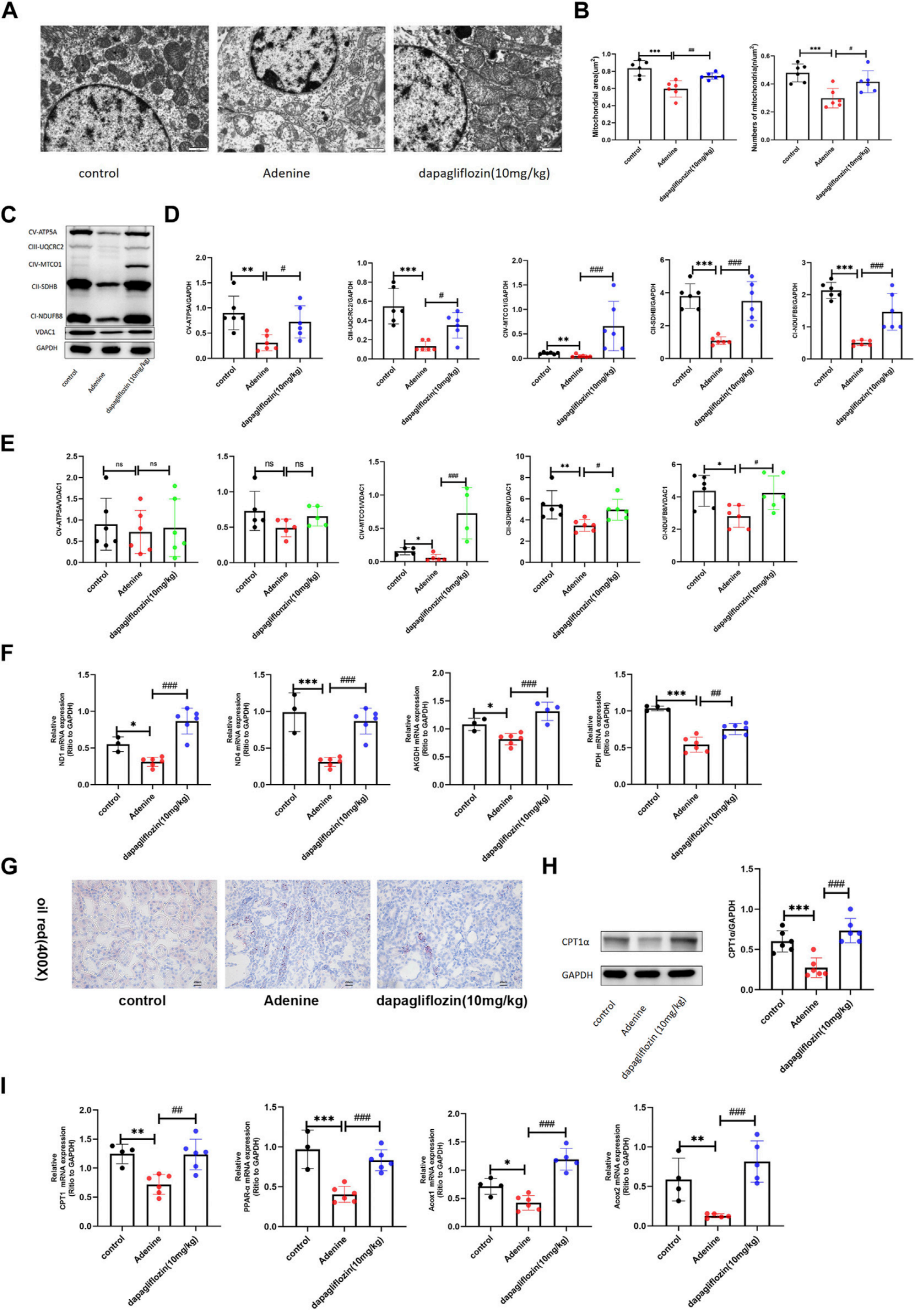
Dapagliflozin increased the levels of mitochondrial metabolism and fatty acid oxidation in 0.2% adenine diet-fed mice. **(A)** Mitochondrial morphology of renal tubular epithelial cells was examined using transmission electron microscopy. Scale bars, 1 μm. **(B)** Mitochondrial areas and amount in renal tubular cell of each group. **(C–E)** Western blot showing the expression of mitochondrial respiratory chain complexes (oxphos) in the renal tissue of each group. **(F)** Real time PCR showing the expression of mitochondrial metabolism-related genes ND1, ND4, AKGDH, and PDH in the renal tissue of each group. **(G)** Renal oil red staining of each group. Scale bars, 20 μm. **(H)**Western blot showing the expression of CPT-1α of in the renal tissue of each group. **(I)** Real time PCR showing the expression of fatty acid oxidation-related genes CPT1, PPAR-α, ACOX1, and ACOX2 in the renal tissue of each group. Values are means ± SD.*p < 0.05 adenine diet group compared with the control group; **p < 0.01 adenine diet group compared with the control group; ***p < 0.001 adenine diet group compared with the control group; ^#^ *p* < 0.01 dapagliflozin treatment group compared with the adenine diet group; ^##^ *p* < 0.01 dapagliflozin treatment group compared with the adenine diet group; ^###^ *p* < 0.001 dapagliflozin treatment group compared with the adenine diet group.

Transcriptomic GO and KEGG enrichment analyses suggested that both mitochondrial metabolism and fatty acid oxidation levels were significantly downregulated in the kidneys of mice fed a 0.2% adenine diet compared with mice in the control group. Mitochondrial metabolism and fatty acid oxidation are important in the occurrence and development of renal injury and fibrosis (Quadri et al., 2019; Console et al., 2020), therefore, we explored the effect of dapagliflozin on these two aspects. First, we examined mitochondrial morphology using electron microscopy. In comparison to control mice, mitochondria with degenerative changes, including disruption of the mitochondrial membrane and matrix swelling, were observed in 0.2% adenine diet-fed mice, and these changes were significantly improved by dapagliflozin treatment (Figure 4A). In addition to the structure of mitochondria, the area and number of mitochondria in renal tubular were decreased in 0.2% adenine diet-fed mice, and the treatment of dapagliflozin could maintain the area and number of mitochondria in renal tubular cell (Figure 4B). Furthermore, mitochondrial respiratory chain complexes regulate mitochondrial oxidative phosphorylation (OxPhos), which is an important part of mitochondrial energy metabolism, like the tricarboxylic acid (TCA) cycle (Edmond, 2009). In comparison to the control mice, the expression of all complexes was decreased in 0.2% adenine diet-fed mice, and dapagliflozin attenuated the reduction of all complexes (Figures 4C, D), partly due to the maintenance of the amount of mitochondria (Figure 4E). Moreover, we used real time PCR to confirm whether the mitochondrial metabolism-related genes, such as ND1, ND4, AKDGH, and PDH, the mitochondrial DNA that encode oxphos-related protein, were downregulated in 0.2% adenine diet mice, and dapagliflozin significantly upregulated the mRNA levels of these genes (Figure 4F). Except for up-regulating the expression of mitochondrial DNA, the RNA-Seq Transcriptomic results shown dapagliflozin also upregulated the expression of nuclear DNA (Supplementary Figure S1). Fatty acid oxidation (FAO) mainly occurs in mitochondria; therefore, mitochondrial dysfunction often leads to the downregulation of fatty acid oxidation (Console et al., 2020). Oil red staining of the mouse kidney tissue showed that lipid deposition in the renal tubules of the mouse kidney was obvious in the 0.2% adenine diet-fed mice compared with control mice, and dapagliflozin reduced lipid deposition in the tubules (Figure 4G). Given that CPT1-α is a key enzyme in fatty acid oxidation (Miguel et al., 2021), we investigated its expression further. Western blotting showed that the expression of CPT1-α was downregulated in the mice that were fed a 0.2% adenine diet, and it was significantly upregulated by dapagliflozin treatment (Figure 4H). The results of real time PCR showed that genes related to fatty acid oxidation, such as CPT1-α, PPAR-α, Acox1, and Acox2, were downregulated in the 0.2% adenine mice, and dapagliflozin attenuated these reductions (Figure 4I).

### 3.5 Dapagliflozin relieved inflammation and oxidative stress in the kidneys of mice fed with 0.2% adenine

**FIGURE 5:**
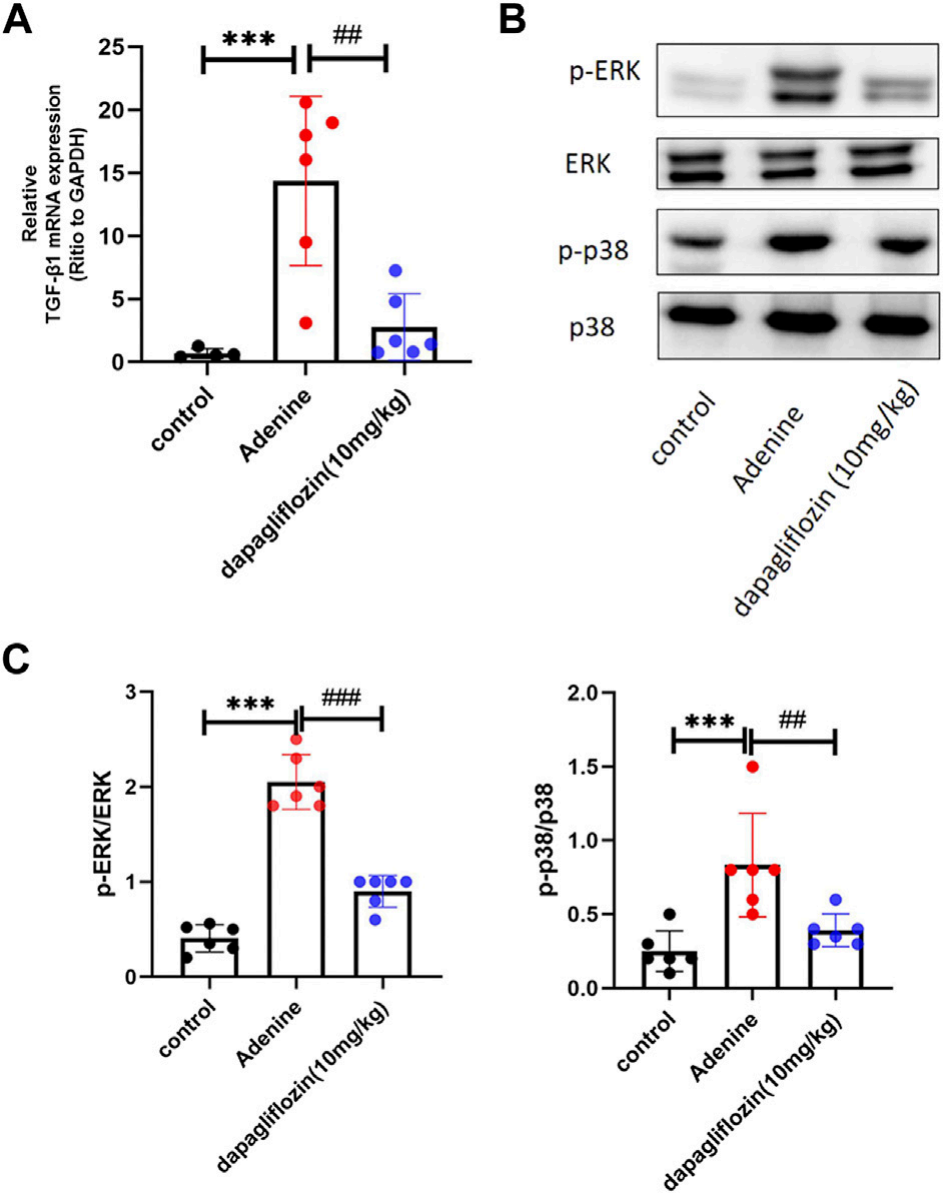
Dapagliflozin alleviated inflammation and increased antioxidant proteins in 0.2% adenine diet-fed mice. **(A)** Real time PCR showing the expression of IL-1β, IL-6, TNF-α, cxcl-1, and mcp-1 in the renal tissue in each group. **(B)** The activity of SOD and content of GSH in the renal tissue of each group. **(C)** The content of MDA in the renal tissue of each group. Values are means ± SD. **p < 0.01 adenine diet group compared with the control group; ***p < 0.001 adenine diet group compared with the control group; ^##^ *p* < 0.01 dapagliflozin treatment group compared with the adenine diet group; ^###^ *p* < 0.001 dapagliflozin treatment group compared with the adenine diet group.

Inflammation and oxidative stress are important factors in the development of renal fibrosis (Meng et al., 2014; Rayego-Mateos and Valdivielso, 2020). The transcriptomic results also showed that inflammation was significantly activated in the mice fed with a 0.2% adenine diet, whereas it was alleviated when the mice were treated with dapagliflozin. Consistently, our real-time PCR results showed that the expression of inflammatory factors such as IL-1β, IL-6, cxcl-1, TNF-α, and MCP-1 was increased in the mice fed with a 0.2% adenine diet compared with control mice, and dapagliflozin treatment reduced their expression (Figure 5A). We also detected SOD activity, GSH and MDA contents in the renal tissue of mice in each group. The results showed that the activity of SOD and content GSH in the kidneys of mice fed with 0.2% adenine decreased, but significantly increased after treatment with dapagliflozin (Figure 5B). The content of MDA increased in the kidneys of mice fed with 0.2% adenine decreased, but decreased after treatment with dapagliflozin (Figure 5C).

### 3.6 Dapagliflozin inhibited TGF-β1/MAPK signaling pathways in the kidneys of mice fed a 0.2% adenine diet

**FIGURE 6:**
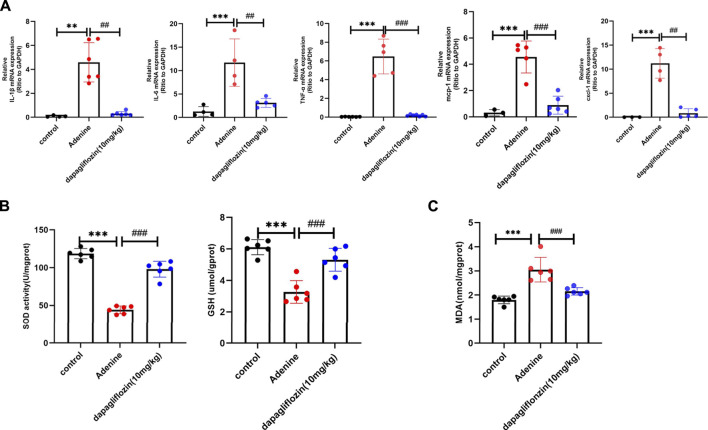
Dapagliflozin inhibited the activation of TGF-β1/MAPK pathway in 0.2% adenine diet-fed mice. **(A)** Real time PCR showing the expression of TGF-β1 in the renal tissue of each group. **(B, C)** Western blot showing the expression of p-ERK and p-P38 in each group. Values are means ± SD. ***p < 0.001 adenine diet group compared with the control group; ^##^ *p* < 0.01 dapagliflozin treatment group compared with the adenine diet group; ^###^ *p* < 0.001 dapagliflozin treatment group compared with the adenine diet group.

TGF-β1 is considered a key driver of renal fibrosis (Meng et al., 2016). Downstream of TGF-β, the MAPK (ERK, JNK, and p38) signaling pathways are overactivated and promote the EMT process in renal fibrosis (Rhyu et al., 2005; Hung et al., 2016). Therefore, we explored whether dapagliflozin affected the TGF-β1/MAPK pathway. Real-time PCR confirmed that TGF-β1 was upregulated when the mice were fed a 0.2% adenine diet, and dapagliflozin reduced its expression (Figure 6A). Western blotting showed that the levels of p-ERK and p-P38 were upregulated in mice fed a 0.2% adenine diet and downregulated by dapagliflozin treatment (Figures 6B, C).

### 3.7 Dapagliflozin inhibits the expression of fibrotic proteins and relieved oxidative stress *in vitro*

**FIGURE 7:**
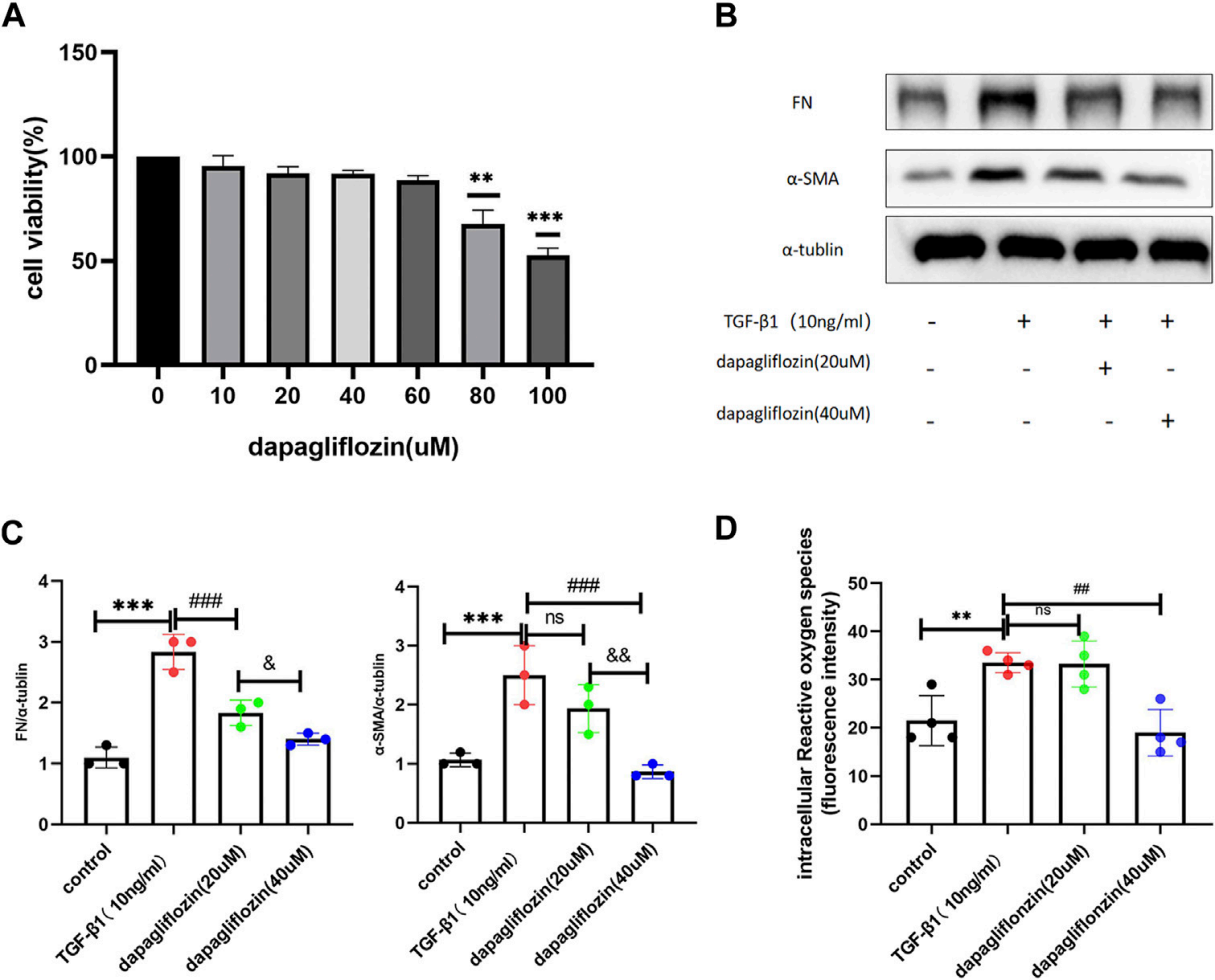
Dapagliflozin reduced the expression of renal fibrosis-related markers in HK2 cells stimulated by TGF-β1. **(A)** CCK8 was used to evaluate the toxicity of dapagliflozin to normal cells. **(B, C)** Western blot showing the expression of FN and α-SMA in HK2 cells in each group. **(D)** The ROS content in HK2 in each group. ***p < 0.001 TGF-β1 stimulated group compared with the control group; Values are means ± SD. ^###^ *p* < 0.001 dapagliflozin treatment group compared with TGF-β1 stimulation group; and *p* < 0.05 20 uM dapagliflozin treatment group compared with 40 uM dapagliflozin treatment group; and *p* < 0.01 20 uM dapagliflozin treatment group compared with the 40 uM dapagliflozin treatment group.

In order to determine the toxicity of dapagliflozin to normal cells, we incubated HK2 cells with dapagliflozin at differing concentrations (0–100 μM) for 24 h. Cell viability was tested using CCK8 reagent (Figure 7A). We then selected the concentrations (20 and 40 μM) that had no apparent effect on normal cell viability for further research. The results suggested that both concentrations of dapagliflozin could significantly inhibit the expression of FN and α-SMA in HK2 cells under stimulation by TGF-β1 (10 ng/mL), and a higher concentration (40 μM) had a more pronounced effect (Figures 7B, C). To further confirm the effects of dapagliflozin, we observed the effects of dapagliflozin *in vitro* model of kidney oxidative stress (H2O2-induced HK-2 cells). The results suggested the ROS level was increased in H2O2-induced HK-2 cells compared to the control group. Treatment with dapagliflozin (40 μM) lowered the level of ROS in HK-2 cells stimulated with H2O2 (Figure 7D).

### 3.8 Dapagliflozin inhibited the TGF-β1/MAPK signaling pathway and attenuated the reduction of mitochondrial respiratory chain complexes in HK2 cells

**FIGURE 8:**
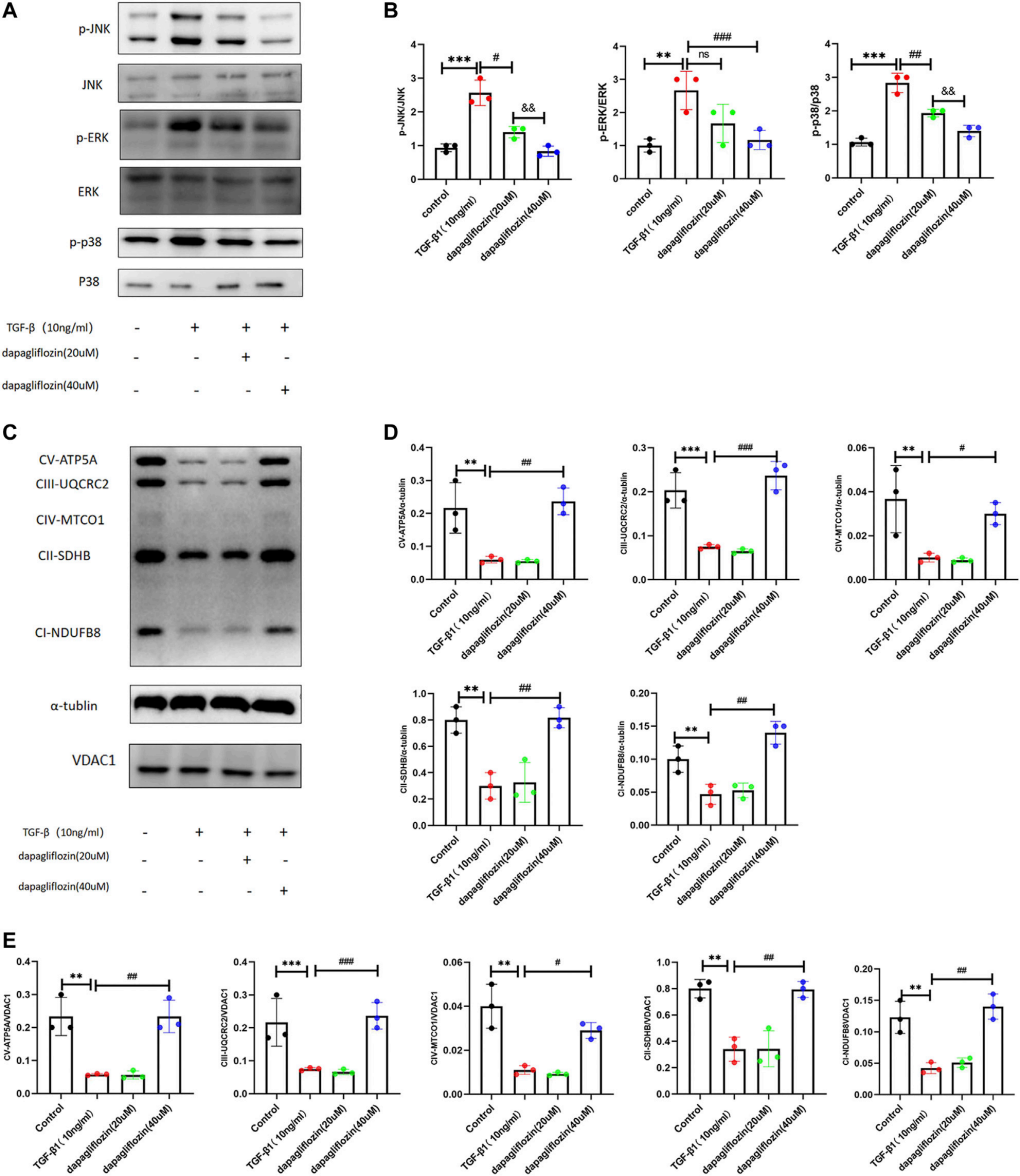
Dapagliflozin inhibited activation of the TGF-β1/MAPK pathway and attenuated the reductions of mitochondrial respiratory chain complexes in HK2 cells stimulated by TGF-β1. **(A, B)** Western blot showing the expression of p-JNK, p-ERK, and p-P38 in HK2 cells in each group. **(C–E)** Western blot showing the expression of mitochondrial respiratory chain complexes (oxphos) in HK2 cells in each group. Values are means ± SD. **p < 0.01 TGF-β1 stimulated group compared with the control group; ***p < 0.001 TGF-β1 stimulated group compared with the control group; #*p* < 0.05 dapagliflozin treatment group compared with the TGF-β1 stimulated group; ^##^ *p* < 0.01 dapagliflozin treatment group with the TGF-β1 stimulated group compared; ^###^ *p* < 0.001 dapagliflozin treatment group compared with the TGF-β1 stimulated group; and *p* < 0.01 20 μM dapagliflozin treatment group compared with the 40 μM dapagliflozin treatment group.

As for the therapeutic group, HK2 cells were incubated with 20 and 40 μM dapagliflozin for 24 h, and 10 ng/mL TGF-β1 was administered to both the model group and the therapeutic groups for 30 min. The cell protein was then harvested for further detection. As seen in Figures 8A, B, the levels of p-JNK, p-ERK, and p-P38 were higher than those in the control group, whereas when treated with dapagliflozin, their expression was much lower, and a higher concentration (40 μM) resulted in a greater reduction in expression. We also found that the expression of mitochondrial respiratory chain complexes decreased in HK2 cells stimulated by TGF-β1, and dapagliflozin (40 μM) increased the level of the complexes (Figures 8C–E).

## 4 Discussion

Renal fibrosis is a common pathological feature of progressive CKD (Meng et al., 2016), for which the clinical management remains challenging. This is partly due to the lack of effective drugs. DN as an important cause of CKD, and some drugs treated with diabetes like SGLT2 inhibitors have shown renal protective effects (Sharma et al., 2017; Kelly et al., 2019), as a typical drug of SGLT2 inhibitors, dapagliflozin’s renal protective effects on CKD have been demonstrated in patients with and without diabetes (Kurata and Nangaku, 2022)*,* and it has also displayed antirenal fibrosis activities in animal models induced by unilateral ureter obstruction and alcohol damage (Wu et al., 2021; Xuan et al., 2021; Liu et al., 2022). In this study, we extended this model to the setting of renal fibrosis induced by a 0.2% adenine diet. By protecting renal function, attenuating renal pathological changes, and reducing the expression of fibrosis-related proteins, including Collagen I, Vimentin, and α-SMA, we confirmed that dapagliflozin had an antifibrotic effect in a 0.2% adenine diet model. Specifically, dapagliflozin sustained mitochondrial integrity and functions, maintained the amount of mitochondria, returned FAO levels, reduced inflammation and oxidative stress, and inhibited the TGF-β1/MAPK signaling pathway in renal tissue.

TGF-β1 is considered a master regulator of epithelial-mesenchymal transition (EMT) and ECM accumulation and, consequently, a potential key driver of renal fibrosis (Meng et al., 2016). As additional downstream pathways of TGF-β, the MAPK (ERK, JNK, and p38) signaling pathways are overactivated and further promote the EMT process in renal fibrosis (Rhyu et al., 2005; Hung et al., 2016). There were some evidences show that in the body of mice fed with 0.2% adenine diet, the excessive adenine would lead to its metabolites (2,8-DHA crystals) deposited in the renal tubules, resulting in tubular injury (Klinkhammer et al., 2020). Then the classic fibrotic pathway MAPK activated, the level of phosphorylation of p38, ERK and JNK increased in adenine-induced renal fibrosis animal model (Rhyu et al., 2012; Tang et al., 2021; Zhou et al., 2021; Ha et al., 2022; Kim et al., 2022). This study provides the first evidence that dapagliflozin could reduce the levels of p-ERK, and p-P38 in renal fibrosis in 0.2% adenine diet-fed mice. *In vitro*, dapagliflozin reduced the levels of p-JNK, p-ERK, and p-P38 in HK2 cells stimulated by TGF-β1. These results suggest that dapagliflozin can inhibitor the activation of TGF-β1/MAPK pathway.

Mitochondria plays an essential role in kidney health, and mitochondrial dysfunction is involved in the physiological process of renal fibrosis (Braga et al., 2022). Studies have shown the members of MAPK signaling pathway can interact with mitochondria to regulate mitochondrial metabolism (Javadov et al., 2014). The activation of ERK, P38, JNK can lead to mitochondrial dysfunction, attenuate pyruvate dehydrogenase activity in the TCA cycle in renal cells, thereby weakening mitochondrial respiration and ATP production, accumulative evidence reveals that insufficient energy supply can lead to renal damage and fibrosis (Nowak, 2002; Nowak et al., 2006; Zhou et al., 2008; Zhou et al., 2009; Simon and Hertig, 2015; Zhao et al., 2019)*.* Thus, activation of the TGF-β1/MAPK pathway is usually accompanied by dysfunction of the mitochondrial function. Fatty acid oxidation mainly occurs in the mitochondria, and lower FAO levels appear to contribute to renal fibrosis development (Simon and Hertig, 2015). In the kidneys, fatty acids are important substrates of ATP production; they are converted to acyl-CoA by acyl-CoA synthetases and then transported to the mitochondria by FAO enzymes, such as CPT-1 and ACOX1 (Yang et al., 2017). During the TCA cycle, pyruvate, generated *via* glycolysis, is converted into acetyl-CoA by PDH, which is then metabolized to carbon dioxide by enzymes such as CS and AKGDH (Martínez-Reyes and Chandel, 2020). Electrons generated in the TCA cycle undergo oxidative phosphorylation to produce ATP (Martínez-Reyes and Chandel, 2020). Our results show that the mitochondrial respiratory chain, TCA cycle, and fatty acid oxidation may play a role in the progression of renal fibrosis induced by a 0.2% adenine diet. In addition to sustaining mitochondrial integrity and maintained the amount of mitochondria, dapagliflozin preserved the expression of ND1, ND4, AKGDH, PDH and ATP5e, SDHA (Mitochondrial DNA and nuclei DNA encode OxPhos protein), along with the expression of mitochondrial respiratory chain complexes in the 0.2% adenine diet model. Except for its protective effects on mitochondria, dapagliflozin preserved the expression of CPT1-α, PPAR-α, ACOX1, and ACOX2, the key enzymes of FAO in 0.2% adenine diet-fed mice. These results suggest dapagliflozin sustained mitochondrial integrity and functions, maintained the amount of mitochondria, returned FAO levels in mice fed a 0.2% adenine diet, and maybe partly due to the inhibition of the TGF-β1/MAPK pathway.

Mitochondrial dysfunction activates inflammation through the mtDNA-cGAS-STING-NF-κB pathway in renal fibrosis, with higher levels of the proinflammatory cytokines IL-6, TNF-α and IL-1β (Chung et al., 2019). Our results showed that dapagliflozin treatment decreased the expression of IL-1β, IL-6, TNF-α, MCP-1, and CXCL-1 in the 0.2% adenine diet-fed mice. Oxidative stress is not only a result of mitochondrial damage, but also a cause of mitochondrial damage. Morphological abnormalities and loss of function of mitochondria will lead to increased ROS production and decreased antioxidant GSH and SOD levels, whereas imbalance of oxidative products and antioxidants will lead to the development of oxidative stress, triggering the progression of renal fibrosis (Zhang et al., 2021; Braga et al., 2022). Inflammation and oxidative stress were critical aspects of renal fibrosis (Meng et al., 2014; Rayego-Mateos and Valdivielso, 2020). Dapagliflozin reduces inflammation and oxidative stress in the kidneys in UUO animal models (Xuan et al., 2021; Liu et al., 2022). Our study also showed that dapagliflozin increased content of GSH, improved activity of SOD, and decreased the content of MDA in 0.2% adenine diet-fed mice. *In vitro*, we also shown that dapagloflozin reduce the ROS lever in H_2_O_2_-induced HK2-cell. Collectively, our research supports the notion that dapagliflozin alleviates inflammation and prevents oxidative stress as a part of its action in protecting against mitochondrial damage.

SGT2 is a clear target of dapagliflozin, thus, we explored whether it plays a role in renal fibrosis. Our study is the first to identify reduced expression of SGLT2 in mice fed a 0.2% adenine diet, and that dapagliflozin was capable of increasing SGLT2 expression (Supplementary Figure S2). Previous reports that the expression of SGLT2 is also decreased when renal tubules are injured by STZ and ischemia-reperfusion (Brouwers et al., 2013; Nespoux et al., 2020). Therefore, we hypothesized that the destruction of tubular structure by a high-adenine diet is responsible for the decreased expression of SGLT2, which reduces SGLT2 by protecting the structure of the kidney, meaning that the change in SGLT2 expression was secondary to the protective effect of dapagliflozin on the kidney. And the therapeutic effect of dapagliflozin on renal interstitial fibrosis is likely independent of SGLT2 inhibiting. Whether dapagliflozin has a specific target in renal fibrosis requires further investigation.

## 5 Conclusion

This study is the first to demonstrate a significant protective effect of dapagliflozin on high adenine diet-induced renal fibrosis. The underlying mechanism was related to mitochondrial protection due to inhibition of the TGF-β1/MAPK pathway activation, then the mitochondrial protection reduced of inflammation and oxidative stress. This study provides new theoretical and experimental evidence of the therapeutic potential of dapagliflozin in renal fibrosis, and it is worth our further study.

## Data Availability

The raw sequence data reported in this paper have been deposited in the Genome Sequence Archive (Genomics, Proteomics & Bioinformatics 2021) in National Genomics Data Center (Nucleic Acids Res 2022), China National Center for Bioinformation / Beijing Institute of Genomics, Chinese Academy of Sciences (GSA: CRA009959) that are publicly accessible at https://ngdc.cncb.ac.cn/gsa.
