# Supplementary material for: Dapagliflozin alleviates renal fibrosis in a mouse model of adenine-induced renal injury by inhibiting TGF-β1/MAPK mediated mitochondrial damage
Source: Front Pharmacol. 2023 Mar 7;14:1095487. doi: 10.3389/fphar.2023.1095487 (PMC10028454; doi:10.3389/fphar.2023.1095487)
Supplement: Supplementary file 1 [file Table1.pdf]

Supplementary Table S1: List of mice primers used for real time PCR

Genes	sequences	
Col1a1(m)	F:5-agacatgttcagctttgtggac-3	R:5-gcagctgacttcagggatg-3
Col1a3(m)	F:5-aggcaacagtggttctcctg-3	R:5-gacctcgtgctccagttagc-3
FN(m)	F:5-cgtaaattgccccattgagtg-3	R:5-gagggtctgctaacatcactg-3
Vimentin(m)	F:5-ctctctgaggctgctaaccg-3	R:5-gagcaatcctgctctcctcg-3
α-SMA（m）	F:5-gtcccagacatcagggagtaa-3	R:5-tcggatacttcagcgtcagga-3
Mmp7(m)	F:5-ctgccactgtcccaggaag-3	R:5-gggagagttttccagtcatgg-3
ND1(m)	F:5-caccccttatcaacctcaa-3	R:5-atttgtttctgcgagggttg-3
ND4(m)	F:5-attattattacccgatgagggaacc-3	R:5-attaagatgagggcaattagcagt-3
AKGDH(m)	F:5-gtttcttcaaacgtggggttct-3	R:5-gcatgattccaggggtctcaaa-3
PDH(m)	F:5-gaaatgtgaccttcatcggct-3	R:5-tgatccgcctttagctccatc-3
CPT1(m)	F:5-ggtcttctcgggtcgaaagc-3	R:5-tcctcccaccagtcactcac-3
PPAR-α	F:5-tttgccaaggctatcccagg-3	R:5-gtcagaacggcttcctca-3
Acox1（m）	F:5-cttggatggtagtccggaga-3	R:5-tggcttcgagtgaggaagtt-3
Acox2(m)	F:5-tcatccaacgtgacccagtg-3	R:5-cagcaaggactctgtcagca-3
IL1-β（m）	F:5-aaggagaaccaagcaacgac-3	R:5-aactgcagactcaaactccac-3
IL-6(m)	F:5-agttgccttcttgggactga-3	R:5-tccacgatttcccagagaac-3
TNF-α（m）	F:5-caggcggtgcctatgtctc-3	R:5-cgatcaccccgaagttcagtag-3
Mcp-1(m)	F:5-taaaaacctggatcggaaccaaa-3	R:5-gcattagcttcagatttacgggt-3
Cxcl-1(m)	F:5-ggcagggattcacttcaaga-3	R:5-acttggggacaccctttagc-3
Sglt2(m)	F:5-ttggtgttggcttgtggtctat-3	R:5-atgttgctggcgaacagaga-3
TGF-β1（m）	F:5-ctcccgtggcttctagtgc-3	R:5-gccttagtttggacaggatctg-3
GAPDH(m)	F:5-catggccttccgtgttccta-3	R:5-cctgcttcaccaccttcttgat-3
