# Supplementary material for: Dapagliflozin alleviates renal fibrosis in a mouse model of adenine-induced renal injury by inhibiting TGF-β1/MAPK mediated mitochondrial damage
Source: Front Pharmacol. 2023 Mar 7;14:1095487. doi: 10.3389/fphar.2023.1095487 (PMC10028454; doi:10.3389/fphar.2023.1095487)
Supplement: Supplementary file 2 [file Image2.pdf]

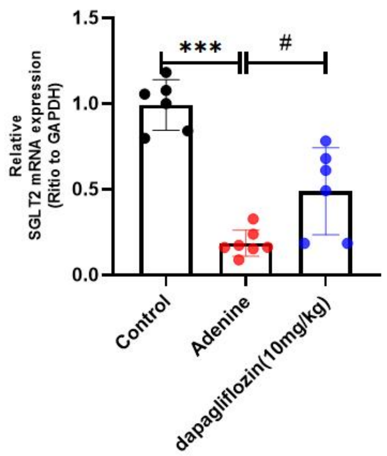

Supplementary Figure S2. Effect of dapagliflozin on SGLT2 in renal tissue of 0.2% adenine-fed mice.The level of SGLT2 mRNA in the kidneys of 0.2% adenine-fed mice decreased, and increased after treatment withdapagliflozin
