# Supplementary material for: Dapagliflozin alleviates renal fibrosis in a mouse model of adenine-induced renal injury by inhibiting TGF-β1/MAPK mediated mitochondrial damage
Source: Front Pharmacol. 2023 Mar 7;14:1095487. doi: 10.3389/fphar.2023.1095487 (PMC10028454; doi:10.3389/fphar.2023.1095487)
Supplement: Supplementary file 3 [file Image1.pdf]

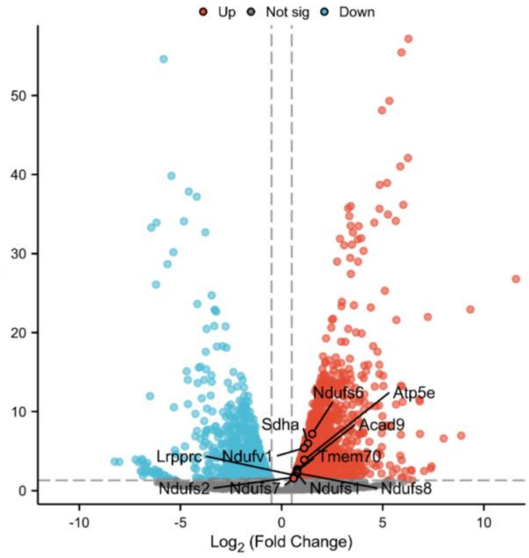

Supplementary Figure S1. Effect of dapagliflozin on expression of nuclear DNA which encode oxphos protein in renal tissue of 0.2% adenine-fed mice(Adenine VS Dapagliflozin). Compare to adenine- fed mice, treated with dapagliflozin up-regulated the expression the nuclear DNA(the red area in the figure).
